# Role of microcirculatory monitoring in polytraumatic patients

**DOI:** 10.1186/cc9870

**Published:** 2011-03-11

**Authors:** A Donati, S Loggi, R Domizi, MR Lombrano, V Fiori, A Valentini, MC Melia, C Scorcella, A Carsetti, P Pelaia

**Affiliations:** 1 Università Politecnica delle Marche, Ancona, Italy

## Introduction

Seventy-five percent of ICU mortality after the first 72 hours following major trauma is due to multiple organ dysfunction syndrome (MODS) [1]. How to follow this evolution is not completely understood yet and new parameters are still needed. The aim of this study was to evaluate the effects of polytrauma on sublingual microcirculation and to search correlations among it, Sequential Organ Failure Assessment (SOFA) score and biochemical markers and to use these factors for monitoring patients [2].

## Methods

This prospective study included 12 patients. Sublingual microcirculation has been registered using sidestream dark field imaging and analysed with AVA software, searching for indices of vessel density, perfusion and type of flow. For each patient, SOFA parameters, hemocoagulation indices, cytonecrosis criteria and hypoperfusion measures have been evaluated at admission and every 48 hours, for a minimum of 96 hours, and correlation between these and microcirculatory parameters has been researched. We then evaluated the discriminating capacity of these parameters versus microcirculatory indices, calculating the area under the ROC curve.

## Results

**Figure 1:**
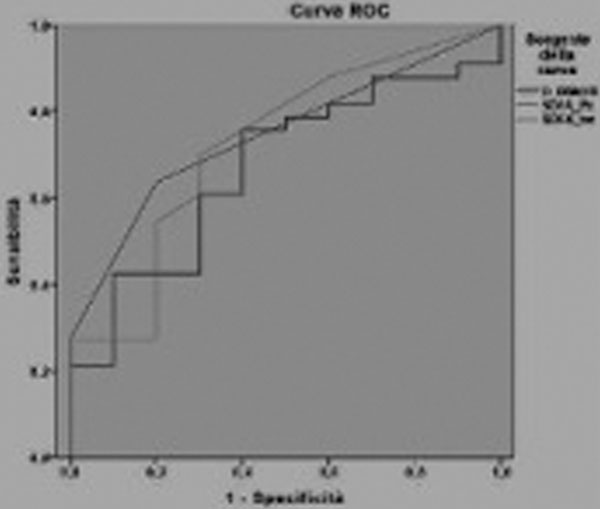
**ROC curve: discriminating capacity of D-dimer, SOFA-Plt, and SOFA-tot versus PVD**.

**Figure 2:**
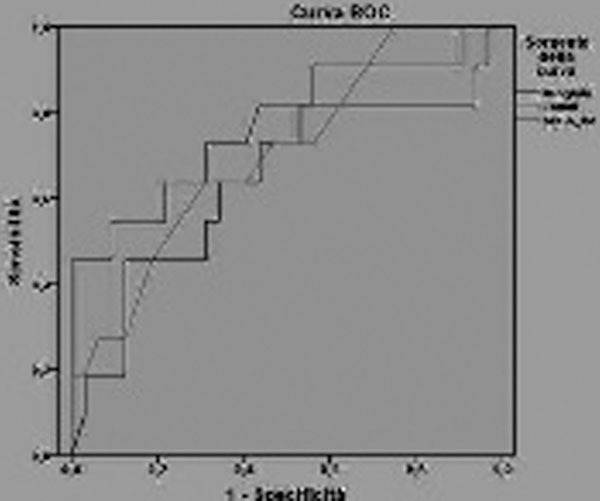
**ROC curve: discriminating capacity of myoglobin, lactate and tot-SOFA versus MFIs**.

No correlation was found between microcirculatory indices and the others. The following parameters had good discriminating capacity: SOFA-platelets (area = 0.745), total-SOFA (0.724) and D-dimer (0.670) for perfused vessel density (PVD) values; Hb (0.693) and SOFA platelets (0.714) for total vessel density (TVD); myoglobin (0.680), lactate (0.732) and total-SOFA (0.703) for microcirculatory flow index (MFI). See Figure 1 and 2.

## Conclusions

Even in polytrauma, microcirculatory dysfunction is important for MODS appearance, and its analysis (PVD, TVD, De Backer score, MFIs) can help to evaluate this evolution, according to biochemical markers and severity index: joined with macrohaemodynamic indices, they allow one to better investigate organ features.
